# Alveolar fluid clearance in healthy pigs and influence of positive end-expiratory pressure

**DOI:** 10.1186/cc8914

**Published:** 2010-03-16

**Authors:** Manuel García-Delgado, Ángel Touma-Fernández, Virginia Chamorro-Marín, Antonio Ruiz-Aguilar, Eduardo Aguilar-Alonso, Enrique Fernández-Mondéjar

**Affiliations:** 1 Department of Intensive Care Medicine, "Virgen de las Nieves" University Hospital, Avda. Fuerzas Armadas, 2, 18014 Granada, Spain; 2 Department of Anesthesiology, "Virgen de las Nieves" University Hospital, Avda. Fuerzas Armadas, 2, 18014 Granada, Spain; 3 Experimental Surgery Laboratory, "Virgen de las Nieves" University Hospital, Avda. Fuerzas Armadas, 2, 18014 Granada, Spain

## Abstract

**Introduction:**

The objectives were to characterize alveolar fluid clearance (AFC) in pigs with normal lungs and to analyze the effect of immediate application of positive end-expiratory pressure (PEEP).

**Methods:**

Animals (n = 25) were mechanically ventilated and divided into four groups: small edema (SE) group, producing pulmonary edema (PE) by intratracheal instillation of 4 ml/kg of saline solution; small edema with PEEP (SE + PEEP) group, same as previous but applying PEEP of 10 cmH_2_O; large edema (LE) group, producing PE by instillation of 10 ml/kg of saline solution; and large edema with PEEP (LE + PEEP) group, same as LE group but applying PEEP of 10 cmH_2_O. AFC was estimated from differences in extravascular lung water values obtained by transpulmonary thermodilution method.

**Results:**

At one hour, AFC was 19.4% in SE group and 18.0% in LE group. In the SE + PEEP group, the AFC rate was higher at one hour than at subsequent time points and higher than in the SE group (45.4% vs. 19.4% at one hour, *P *< 0.05). The AFC rate was also significantly higher in the LE + PEEP than in the LE group at three hours and four hours.

**Conclusions:**

In this pig model, the AFC rate is around 20% at one hour and around 50% at four hours, regardless of the amount of edema, and is increased by the application of PEEP.

## Introduction

Resorption of alveolar fluid is the key to resolving pulmonary edema, and considerable research efforts have focused in recent years on the mechanisms that underlie alveolar clearance [1-3]. Active ion transport is the main mechanism involved in the removal of fluid from distal air spaces of the intact lung. Other catecholaminergic and non-catecholaminergic mechanisms have been related to alveolar edema clearance under pathological conditions [4]. The rate of pulmonary edema clearance has been measured in many animal species [5-12] but remains unknown in pigs, despite the common use of this animal in experimental research. The methods used to study alveolar fluid clearance (AFC) are frequently invasive, such as protein alveolar concentration [13] or isotope-labeled albumin [14] analysis, or are destructive, as with the gravimetric method [15]. This last technique is considered the gold standard by many authors, but it does not detect variations in extravascular lung water (EVLW) over time because it only yields one data point. In contrast, multiple EVLW measurements can be made with the transpulmonary thermodilution technique, enabling study of the time course or clearance profile of the fluid in a simple manner.

Preservation of the capacity to remove alveolar fluid has been associated with a decrease in morbidity and mortality in patients with acute respiratory distress [16]. Therefore, strategies aimed at accelerating or improving pulmonary edema clearance may be beneficial to resolve edema [2]. However, the effect on the AFC rate of positive end-expiratory pressure (PEEP), a common clinical maneuver, has yet to be elucidated. The objectives of this study were to characterize the alveolar edema clearance profile in pigs with normal lungs and to test the hypothesis that the immediate application of PEEP increases the AFC rate.

## Materials and methods

The study was approved by the ethical committee of our hospital, and the animals were managed according to Spanish norms for the protection of experimental animals (Royal Decree 1201/2005).

### Animal preparation and general experimental protocol

Twenty-five mixed-breed pigs weighing 30 ± 5 kg were premedicated with intramuscular injection of ketamine (10 mg/kg) and azoperone (5 mg/kg). After canalization of an ear vein, anesthesia was induced by the intravenous injection of atropine (1 mg), ketamine (2 mg/kg), and fentanyl (0.15 mg). A tracheotomy was performed *via *midline incision, immediately followed by intubation with a cuffed tube (6.5 mm internal diameter). The pigs were then connected to mechanical ventilation at a tidal volume of 10 ml/kg, respiratory rate of 20 breaths/minute, inspiratory:expiratory ratio of 1:2, and FiO_2 _of 0.6. Anesthesia was maintained with a continuous infusion of ketamine (20 mg/kg/h) and atracurium (1 mg/kg/h), administering supplementary boluses of fentanyl and atracurium when necessary. The animals received a continuous infusion of 0.9% saline solution (3 ml/kg/h) throughout the experiment.

A double-lumen 7-Fr catheter (CV-17702, Arrow, Erding, Germany) was placed in the left external jugular vein, and a 5-Fr thermistor-tipped catheter (PV-2015L13, Pulsion Medical Systems, Munich, Germany) was advanced into the descending aorta and connected to a PICCO^® ^computer (Pulsion Medical Systems) for EVLW determinations.

Baseline measurements were made after a 30-minute period of stable heart rate and systemic blood pressure. Immediately afterwards, alveolar edema was induced by instillation of saline solution via the tracheal tube. Only two or three respirations were permitted between introduction of the saline solution and the second measurement (Time 0), and these were strictly scrutinized to ensure that no liquid escaped through the tracheal tube. Thereafter, parameters were also measured at 60, 120, 180, and 240 minutes.

### Specific experimental protocol

In the small-edema (SE) group (n = 10), edema was induced by intratracheal instillation of 4 ml/kg of saline solution. In the large-edema (LE) group (n = 5), edema was induced by intratracheal instillation of 10 ml/kg of saline solution. In the small-edema with PEEP (SE + PEEP) group (n = 5), edema was induced by intratracheal instillation of 4 ml/kg of saline solution, applying PEEP of 10 cm H_2_O immediately after the first determination of EVLW (before time 0). In the large-edema with PEEP (LE + PEEP) group (n = 5), edema was induced by intratracheal instillation of 10 ml/kg saline solution, applying PEEP of 10 cm H_2_O immediately after the first determination of EVLW (before time 0).

### Measurements

#### Extravascular lung water

EVLW was determined by infusing a 10 ml bolus of saline solution at <8°C *via *the central venous catheter. The thermodilution curve was recorded using the thermodilution catheter in the aorta, and EVLW data were collected from the PICCO^® ^monitor, considering the mean of three measurements as the EVLW value.

#### Alveolar fluid clearance

Calculation of the AFC was based on the EVLW measurements obtained by transpulmonary thermodilution, subtracting EVLW values at time 0 from baseline values to obtain the added fluid (F_added_). The AFC for each time period is expressed as a percentage of the F_added _value. Hence, for time n:

Differences in clearance rates were recorded as a function of the application or not of PEEP and as a function of the amount of saline solution instilled.

#### Gas exchange and airway pressure

Arterial blood gas samples were immediately analyzed with an ABL-700 blood gas analyzer (Radiometer, Copenhagen, Denmark), determining PaO_2 _values. Peak and plateau airway pressures were also recorded.

#### Hemodynamic parameters

Blood pressures and cardiac output were recorded every 60 minutes by means of the PiCCO^® ^monitor.

#### Statistical analysis

EVLW and hemodynamic and respiratory parameters are expressed as means and standard deviation. AFC rates are expressed as the percentage of fluid cleared up to the measurement time point. A repeated-measures analysis of variance (ANOVA) was used to analyze changes in variables over time. The Mann Whitney U-test for independent samples was used to compare among groups. For all tests, *P *< 0.05 was considered statistically significant.

## Results

### Time course of EVLW

EVLW values at each time point are summarized in Table 1. Baseline values did not significantly differ among groups and markedly and significantly increased after the intratracheal instillation of saline solution, followed by a decrease that varied among groups.

**Table 1:** Extravascular lung water and respiratory and hemodynamic parameters.

	Baseline	0	60 minutes	120 minutes	180 minutes	240 minutes
**EVLW (ml)**						
SE	286 (72) ^a^	421 (93)	395 (81)	356 (57)	346 (58)	344 (63)
LE	225 (30) ^a^	458 (42)	415 (34)	387 (27)	354 (40)	331 (45)
SE+PEEP	309 (80) ^a^	446 (64)	383 (60)	371 (70)	367 (75)	363 (73)
LE+PEEP	269 (37) ^a^	491 (56) ^b^	436 (28) ^b^	389 (54)	366 (43)	349 (46)
						
**PaO_2_/FiO_2_**						
SE	346 (148) ^b^	167 (91)	193 (95)	204 (121)	216 (134)	221 (141)
LE	416 (128) ^b^	100 (36)	96 (49)	118 (44)	169 (62)	183 (54)
SE+PEEP	269 (156)	420 (74) ^c^	432 (89) ^c^	424 (107) ^c^	403 (127) ^c^	425 (121) ^c^
LE+PEEP	437 (69)	202 (93) ^d^	262 (144) ^d^	401 (141) ^d^	505 (29) ^d^	539 (27) ^d^
						
**Pplat (mmHg)**						
SE	11.8 (2.7) ^a^	15.3 (1.7)	15.4 (2.2)	15.3 (1.9)	15.1 (2.2)	14.9 (2.0)
LE	10.8 (2.3) ^a^	17.1 (1.5)	16.2 (3.0)	15.2 (1.9)	15.2 (2.3)	15.4 (2.4)
SE+PEEP	14.8 (3.4) ^a^	23.4 (3.1) ^e^	23.2 (1.6) ^e^	23.2 (1.7) ^e^	23.2 (1.7) ^e^	23.1 (1.8) ^e^
LE+PEEP	14.4 (4.5) ^a^	27.4 (7.4) ^f^	26.6 (4.9) ^f^	26.9 (4.7) ^f^	25.8 (4.3) ^f^	25.8 (4.7) ^f^
						
**MAP(mmHg)**						
SE	68.2 (9.5)	68.2 (9.3) ^a^	76.9 (12.3)	82.7 (10.5)	84.3 (9.6)	87.3 (10.8)
LE	77.2 (10.7)	77.0 (5.1)	80.2 (6.8)	77.4 (5.7)	81.2 (10.7)	82.4 (12.8)
SE+PEEP	59.0 (8.3)	61.7 (5.5)	69.8 (8.2)	75.0 (9.6)	76.2 (12.7)	77.4 (13.3)
LE+PEEP	70.4 (13.8) ^g^	52.4 (10.5)	63.2 (5.4)	66.8 (4.8)	64.4 (4.8)	64.7 (5.0)
						
**CO (L/min)**						
SE	3.7 (0.9)	3.9 (1.0)	4.5 (1.2)	4.7 (0.9)	4.6 (0.9)	4.5 (0.9)
LE	3.9 (1.2)	3.9 (0.9)	4.6 (1.4)	4.5 (1.2)	4.1 (0.9)	3.9 (0.9)
SE+PEEP	3.9 (0.7)	4.7 (0.8)	4.4 (0.9)	4.2 (0.8)	4.3 (0.9)	4.1 (1.0)
LE+PEEP	3.8 (1.1) ^b^	3.0 (1.0) ^d^	3.9 (0.8) ^d^	3.7 (0.9) ^d^	3.4 (0.8) ^d^	3.2 (0.7) ^d^

Data are expressed as mean (SD).

a Statistically significant differences with subsequent time points (*P *< 0.01).

b Statistically significant differences with subsequent time points (*P *< 0.05).

c Statistically significant differences between SE and SE + PEEP groups (*P *< 0.05).

d Statistically significant differences between LE and LE + PEEP groups (*P *< 0.05).

e Statistically significant differences between SE and SE + PEEP groups (*P *< 0.01).

f Statistically significant differences between LE and LE + PEEP groups (*P *< 0.01).

g Statistically significant differences between baseline and time 0 (*P *< 0.05). CO, cardiac output; EVLW, extravascular lung water; LE, large edema; LE + PEEP, large edema + positive end-expiratory pressure; MAP, mean arterial pressure; Pplat, plateau pressure; SE, small edema; SE + PEEP, small edema + positive end-expiratory pressure.

### Alveolar fluid clearance

**Figure 1:**
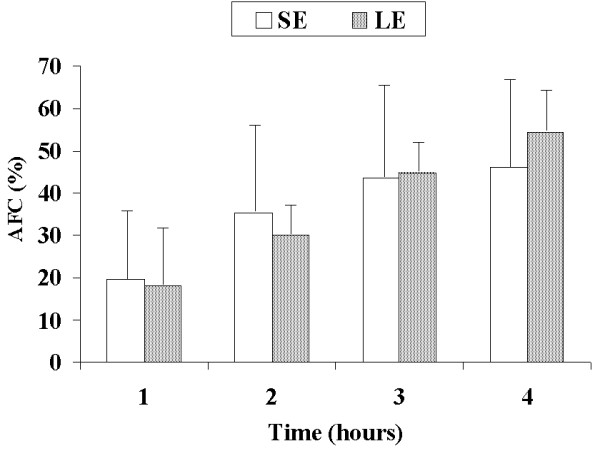
**Comparison of alveolar fluid clearance (percentage with respect to initial edema) between small-edema and large-edema groups**. Each bar represents the mean ± SD.

**Figure 2:**
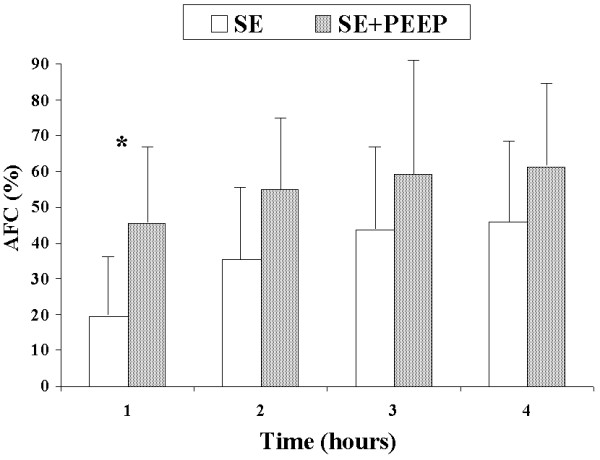
**Comparison of alveolar fluid clearance (percentage with respect to initial edema) between small-edema and small-edema with PEEP groups**. Each bar represents the mean ± SD. *P < 0.05 between groups.

**Figure 3:**
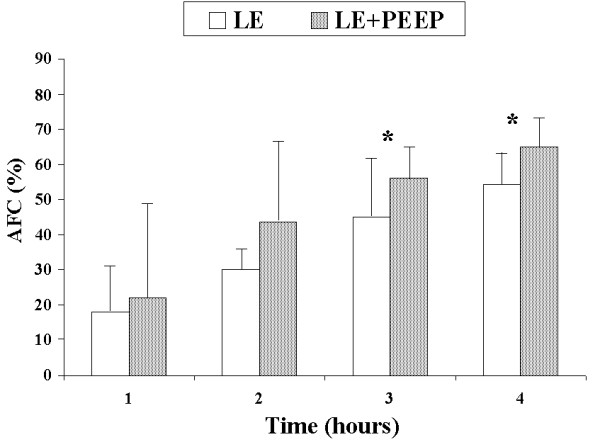
**Comparison of alveolar fluid clearance (percentage with respect to initial edema) between large-edema and large-edema with PEEP groups**. Each bar represents the mean ± SD. *P < 0.05 between groups.

**Figure 4:**
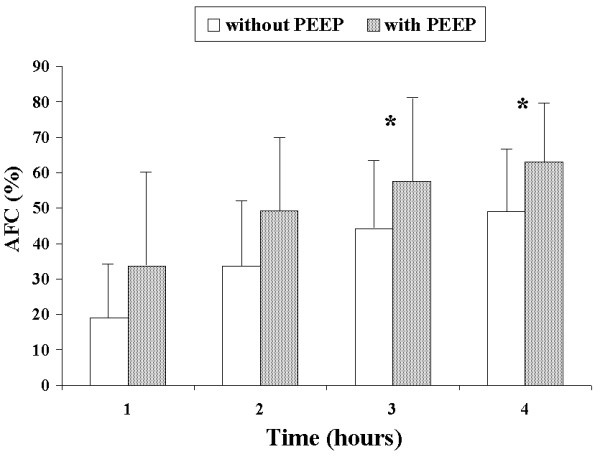
**Comparison of alveolar fluid clearance (percentage with respect to initial edema) between groups with and without PEEP**. Each bar represents the mean ± SD. *P < 0.05 between groups.

AFC rates were similar between the SE and LE groups at one hour (19.4% vs. 18.0%, *P *= 0.7) and four hours (46.0% vs. 54.3%) (Figure 1). PEEP application in the SE + PEEP group produced an early increase in AFC rate, which was significantly higher than in the SE group at one hour (45.4% vs. 19.4%, *P *= 0.04) (Figure 2). The AFC rate was significantly lower in the LE group than in the LE + PEEP group at three hours (44.9% vs. 55.9%, *P *= 0.02) and at the end of the experiment (four hours) (54.3% vs. 65.0% *P *= 0.04) (Figure 3). At four hours, the AFC rate was significantly lower in the two groups without PEEP than in the groups with PEEP (49.0% vs. 63.1%, *P *= 0.01) (Figure 4).

### Respiratory parameters

Oxygenation and airway pressures are shown in Table 1. Immediately after induction of alveolar edema, the PaO_2_/FiO_2 _ratio sharply decreased in all groups except in the SE + PEEP group. Thereafter, oxygenation remained unchanged in the SE + PEEP group and progressively improved in the SE and LE groups, although without reaching pre-instillation levels. The LE + PEEP group showed the greatest increase in oxygenation, which was higher than the pre-instillation level by the end of the experiment. The intratracheal instillation of saline produced a moderate increase in plateau pressure in all groups.

### Hemodynamic parameters

Table 1 also shows the mean cardiac output and systemic blood pressure values, which all remained within physiological ranges and did not significantly differ among the groups.

## Discussion

In this pig model of alveolar edema, an AFC rate of around 20% in the first hour was observed at both edema levels studied (4 ml/kg and 10 ml/kg). Although the absolute amount of liquid resorbed (in ml) was higher in the large-edema group, the AFC rate (in %) was similar among the groups and independent of the amount of edema. After the first hour, the clearance tended to diminish in all groups, which can be attributed to the small amount of alveolar fluid left for resorption. The number of flooded alveoli able to clear fluid would be very low in this situation, and a larger exchange surface area is known to be associated with a higher AFC rate [17]. A further factor in this reduced AFC rate may be a decrease in the level of endogenous catecholamines, due to the lower EVLW and improved arterial oxygenation. Endogenous catecholamines have been related to the AFC rate under experimental conditions of hypovolemia and septic shock in rats [18,19], neurogenic pulmonary edema in dogs [20], and left auricular hypertension in sheep [21]. Nevertheless, their role has yet to be defined, since studies of hydrostatic and lesional pulmonary edema in humans [13,22] found no relationship between endogenous catecholamine levels and the clearance rate. Finally, the decrease in AFC rate in the last hour was probably not due to the physical barrier represented by the accumulation of fluid in the pulmonary interstitium. The animal species in which this has been reported have a higher clearance rate in comparison to pigs [8].

The AFC rate observed in this study is higher than that reported in other animals of similar size, for example, 6% in dogs [7] and 9 to 10% in sheep [6,7] and goats [23], and lower than that in smaller animals, for example, rabbits, guinea pigs, rats, and mice [8-10]. Comparisons with humans are hampered because the initial amount of pulmonary edema in human lung is poorly documented except in studies of *ex-vivo *human lungs [24]. Nevertheless, it has been estimated that humans with intact alveolar epithelium and hydrostatic pulmonary edema have a medium-high AFC rate of 25% per hour [22].

In the small-edema group, PEEP application produced a major and significant increase in the AFC rate during the first hour, with a low resorption rate thereafter. The decline in the AFC rate after the first hour can be explained by the fact that almost half of the alveolar edema had already been cleared, leaving around 70 ml to be resorbed. The initial increase in the AFC in this group can probably be attributed to the larger number of alveoli available to clear the instilled fluid after the PEEP application. It is well known that PEEP application partially restores the residual functional capacity by recruiting new alveoli units and preventing their collapse at the end of the expiration [25]. When the edema was larger (10 ml/kg), the PEEP application also increased the clearance rate but later, with a higher rate only observed after three hours. The weight of the larger amount of edema may have contributed to the alveolar recruitment in this group, increasing the number of alveolar units available for the clearance and reducing the initial effect of PEEP application.

PEEP can produce a fall in cardiac output (CO) especially in situations of hypovolemia. In the group with the larger edema, PEEP application induced a CO decrease that was maintained throughout the experiment, although it was more marked at the first determination with PEEP (time 0). The CO decrease may have resulted from a combination of factors: the limitation of venous return due to the PEEP; and the intratracheal instillation of a larger amount of saline solution, producing a greater increase in plateau pressure and hence a larger reduction in venous return. We cannot rule out that this fall in CO might have caused an underestimation of the EVLW, since transpulmonary thermodilution is perfusion-dependent technique, but we consider that this would only be significant in extreme situations, with a much more marked CO decrease than recorded in our study. No data are currently available to permit calibration of the magnitude of this possible underestimation. However, the fact that EVLW clearance behavior did not differ among the groups suggests that this effect did not have a major impact on our results.

The intratracheal instillation of saline solution induced a fall in oxygenation in the groups without PEEP but not in the groups with PEEP. Introduction of the solution produced an increase in plateau pressure in all groups that was maintained without significant changes throughout the experiment; this increase was greater in the groups with PEEP. The maintenance of plateau pressures could be explained by the presence of PEEP in the latter groups, but a certain improvement in plateau pressures could be expected in the groups without PEEP as the EVLW decreases. The lack of improvement in these groups may be due to a reduction in the residual functional capacity as a result of the four-hour ventilation without PEEP. The fall in PaO_2_/FiO_2 _in the groups without PEEP would support this hypothesis.

We used the intratracheal administration of saline solution as an extremely simple reference method that provides accurate information on EVLW variations. We consider it to be a good choice for detecting EVLW variations over time. However, it may be considered a potential study limitation, since the edema produced by the intratracheal administration of saline solution is not physiological. It is exclusively alveolar and protein-free, whereas the edema in the clinical setting is usually bottom-up and therefore mixed (interstitial and alveolar). Our model is similar to that which could be produced by near-drowning in fresh water. A mixed interstitial and alveolar edema is theoretically easier to detect by the transpulmonary thermodilution method, because the cold vector travels from the vascular space to the interstitial space and then to the alveolar space. However, if the edema is solely alveolar, as in the present case, the more easily detectable interstitial component is absent. Under these conditions, the transpulmonary thermodilution technique appears highly sensitive [26], although we cannot rule out some influence on the results. A further limitation is that our results cannot be extrapolated to injured lungs or larger amounts of alveolar fluid, because we studied healthy lungs in which the alveolar-capillary membrane and resorption mechanisms were considered intact. Thermodilution is a perfusion-dependent technique that does not take non-perfused areas into account, which would have a greater effect in injured than in healthy lungs. Finally, we cannot rule out a methodological bias related to the use of PEEP, since its application could produce an underestimation of EVLW level by a reduction in the perfusion and distribution of the indicator [27]. Nevertheless, we do not believe that this factor affected the present results, since it would also have produced a greater initial clearance in the group with high edema. In fact, various studies have demonstrated that 10 cmH_2_O of PEEP does not produce a significant underestimation of the EVLW [28].

## Conclusions

In conclusion, under the present experimental conditions, the clearance rate in pigs with healthy lungs is around 20% after one hour and around 50% after four, regardless of the amount of edema produced. This is closer to the rate estimated in humans with healthy lungs than has been reported in other animal species. The application of PEEP produces an increase in the clearance rate that occurs earlier when a small amount of alveolar edema is produced.

## Key messages

• Alveolar fluid clearance in pigs with healthy lungs is around 20% after one hour.

• The clearance rate is independent of the amount of saline solution introduced (small or large).

• In small edemas, PEEP application produces an early increase in the alveolar fluid clearance rate.

• The transpulmonary thermodilution method permits the accurate monitoring of extravascular lung water.

## Abbreviations

AFC: alveolar fluid clearance; CO: cardiac output; EVLW: extravascular lung water; LE: large edema; PE: pulmonary edema; PEEP: positive end-expiratory pressure; SE: small edema.

## Competing interests

MGD, ATF, VCM, ARA and EAA declare that they have no competing interests. EFM is a member of Pulsion's Medical Advisory Board.

## Authors' contributions

MGD, ATF and EFM designed the study and drafted the manuscript. MGD, VCM, ARA, and EAA were involved in the animal experiments. MGD and ATF performed the statistical analysis. EFM coordinated the study. All authors read and approved the final manuscript.
